# Navigating complexities: agile digital health initiatives in developing countries

**DOI:** 10.1093/oodh/oqae032

**Published:** 2024-08-20

**Authors:** Indu Bhushan, Kiran Anandampillai, Smisha Agarwal

**Affiliations:** Department of International Health, Johns Hopkins Bloomberg School of Public Health, Baltimore, MD 21205, USA; Department of Information Technology, National Health Authority, Bengaluru, Karnataka 560009, India; Department of International Health, Johns Hopkins Bloomberg School of Public Health, Baltimore, MD 21205, USA; Center for Global Digital Health Innovation, Department of International Health, Johns Hopkins Bloomberg School of Public Health, Baltimore, MD 21205, USA

The intersection of digital technology and healthcare holds profound promise for advancing universal health coverage on a global scale. In recent years, the evolution of digital health has transcended traditional boundaries, offering transformative prospects that are particularly advantageous for developing nations. Unlike their developed counterparts, burdened by entrenched legacy systems and interoperability challenges, developing countries possess a unique advantage in constructing agile and interoperable digital health infrastructures from the ground up. By leveraging insights from the digital health trajectories of more advanced economies, these countries can circumvent outdated paradigms and tailor innovative, sustainable solutions that address their specific healthcare needs and challenges [1].

Mainstreaming digital health in developing countries faces significant hurdles. Severe resource constraints limit the ability to invest in technology and infrastructure. Uneven digital infrastructure creates disparities in access, leaving some regions underserved. A shortage of skilled technical personnel hampers the effective deployment and maintenance of digital health systems. The rapid pace of technological advancement risks obsolescence of newly implemented solutions before they are fully integrated. Nascent regulatory frameworks often struggle to keep up with the fast-evolving digital landscape, leading to gaps in oversight and standardization. Additionally, limited implementation capacities can impede the practical application of digital health innovations, reducing their impact and effectiveness [1, 2].

A crucial issue for policymakers in developing countries is balancing investments in digital health initiatives with the need to expand physical healthcare services. This concern stems from the finite resources available, which must be allocated between upgrading digital tools and expanding traditional healthcare infrastructure. However, empirical evidence indicates that well-designed digital health interventions can actually complement and enhance physical healthcare services rather than detract from them. For instance, digital health tools can improve access to care by facilitating remote consultations, which is particularly valuable in underserved areas. They can also boost the effectiveness and efficiency of healthcare delivery through streamlined data management and better coordination among providers. Moreover, digital systems can enhance accountability and transparency by providing robust tracking and reporting mechanisms. Consequently, while initial investments in digital health may seem daunting, their potential to significantly improve overall healthcare delivery makes them a strategic and beneficial choice [3]. The papers in this special issue address the challenges of integrating digital health solutions into existing healthcare systems and offer practical strategies for overcoming these obstacles. The issue aims to thoroughly document the development and implementation of core digital health frameworks in developing countries. Its primary goal is to promote mutual learning among various stakeholders, facilitating a shared understanding and collaboration to enhance the integration of digital health initiatives.

A Digital Public Infrastructure (DPI) approach, reflected across the papers, is crucial for transforming digital health strategies by creating an interconnected ecosystem that facilitates seamless data exchange across various healthcare systems. DPI refers to the foundational digital systems and services that enable secure, reliable and inclusive access to digital resources. By adopting a DPI approach, countries can overcome the challenges posed by fragmented and siloed health systems, ensuring that critical health data are accessible across platforms and applications.

This approach supports the integration of electronic health records, telemedicine and other digital health tools, leading to improved patient outcomes and more efficient care delivery. The World Health Organization's Global Initiative on Digital Health aims to guide countries in implementing such strategies, recognizing that a robust DPI can bridge gaps between different health systems and foster greater collaboration. Ultimately, DPI enables countries to build more resilient, responsive and equitable health systems in the digital age.

DPIs are akin to roads—they are essential investments, usually made by governments, to create and manage core digital frameworks that enable the broader ecosystem. For example, India is developing DPIs in healthcare through the Ayushman Bharat Digital Mission. These DPIs facilitate the creation of personal health records for every resident, support interoperability in digital health services like appointment booking and telemedicine and streamline interactions between payers and providers for health claims [4]. This approach is proving effective in bringing interoperability to siloed systems, ensuring security and privacy, and leveraging private sector capacity for digital health. It is crucial that DPIs incorporate privacy and security by design, in alignment with the country’s data protection laws [5–7].

Foundational to the conceptualization of architecture for digital health is the importance of engaging local stakeholders from the very beginning of the design process, so that the effectiveness of proposed interventions is evident. This would also further the support of relevant local funders for digital infrastructure [2, 8]. Waiyaiya et al. (2024) argue that a structured and standardized health information exchange system can be built through structured discussions with key stakeholders. They emphasize the critical need for broad stakeholder engagement—from government bodies and private sector entities to academia, investors and implementation partners—to drive forward the digital transformation of healthcare systems [8].

Proliferation of donor-driven, small-scale digital health initiatives, while well-intentioned, has inadvertently contributed to fragmentation and the creation of isolated innovation silos. Addressing these challenges is crucial to prevent the entrenchment of disjointed systems that undermine the broader goal of integrated healthcare delivery. For example, in India there are separate applications used by field level workers for TB, reproductive health care and non-communicable diseases. None of these applications are integrated with each other and have been developed by three different agencies. This fragmented approach has deterred integrated care and increased workload for health workers. Mehl et al. (2023) advocate for a paradigm shift from project-driven approaches to person-centered digital health systems, advocating for the adoption of Open Standards, Technologies, Architectures and Content (full-STAC). This approach represents a progressive step toward fostering interconnected and interoperable digital health ecosystems that can support comprehensive healthcare delivery models in developing countries [7].

Establishing compatible common standards for digital health infrastructure and information is identified as a critical enabler for ensuring seamless interoperability within national borders and across global healthcare systems [9]. Egwar et al. (2024) illustrate the challenges faced by Uganda, where diverse digital health interventions have failed to achieve their potential due to interoperability issues arising from the absence of standardized frameworks. They underscore the urgent need for developing countries that have yet to standardize their health systems to prioritize the adoption of digital health standards. The authors propose a collaborative framework for building consensus on standardized digital health communication infrastructures, emphasizing the importance of context-specific approaches informed by extensive stakeholder consultations [10].

Governance of digital health systems poses a significant challenge in developing countries, where existing institutional capacities are often overstretched, and the technical complexities involved are substantial [1, 5, 6, 11]. The DPI-approach requires Governments to lay out the governance approach for the public infrastructure and craft incentives that lead to adoption of the DPI by the ecosystem. Kijsanayotin et al. (2024) present a comparative analysis of digital health governance models across Thailand and several other countries. They advocate for the establishment of dedicated agencies independent of traditional healthcare ministries, equipped with robust leadership structures, effective interagency collaboration mechanisms and consistent health information standards nationwide. Such initiatives are seen as essential for overcoming governance challenges and ensuring the sustainable implementation of digital health strategies in diverse socio-economic contexts [12].

Tailoring the foundational architecture for digital health to a country's specific socio-economic, cultural, political and technological contexts is crucial for successful implementation [2, 5, 6, 11]. While universal principles like interoperability, privacy and security are essential, they must be adapted to local realities. For example, a federated data storage architecture, which keeps data close to its source, may work well in larger countries with robust infrastructure but could be impractical for smaller, resource-constrained nations where centralized systems might be more feasible. Similarly, adopting a DPI approach for healthcare nationally might pose challenges in countries lacking basic infrastructure, such as reliable personal identification systems or widespread mobile connectivity. In such contexts, the high costs associated with establishing and maintaining digital health systems might necessitate a regional approach to share resources and leverage economies of scale. Countries with significant inequalities and uneven access to IT services must design digital health systems that do not widen the digital divide, ensuring equitable access to healthcare. Moreover, the design and implementation of digital health infrastructure must consider the technical capacity of the country, including the availability of trained personnel and the strength of the private IT sector. By aligning digital health architecture with these contextual factors, countries can create more effective and sustainable health systems.

Looking ahead, the rapid evolution of digital technologies, including artificial intelligence (AI), introduces both unprecedented opportunities and novel complexities for healthcare systems worldwide. AI holds the potential to revolutionize diagnostics, treatment protocols and healthcare delivery models by leveraging vast amounts of data and sophisticated algorithms. However, the deployment of AI in healthcare necessitates robust ethical frameworks, stringent regulatory oversight and equitable access to ensure that these technologies benefit all segments of society. Achieving this balance requires collaborative partnerships among policymakers, healthcare providers, technologists, researchers and community stakeholders. By harnessing collective expertise and adopting a holistic approach, the transformative power of digital health can be effectively harnessed to advance toward the overarching goal of achieving universal health coverage [13].

This special issue contributes timely insights and empirical evidence on the opportunities and challenges associated with the digital transformation of healthcare systems in developing countries. By synthesizing diverse perspectives and experiences, the papers featured here offer valuable lessons and actionable recommendations for policymakers, healthcare practitioners, researchers and other stakeholders committed to leveraging digital technologies to enhance healthcare access, quality and equity on a global scale.
